# Gaming with health misinformation: a social capital-based study of corrective information sharing factors in social media

**DOI:** 10.3389/fpubh.2024.1351820

**Published:** 2024-04-18

**Authors:** Bobo Feng

**Affiliations:** School of Journalism and Media, Chongqing Normal University, Chongqing, China

**Keywords:** social media, social capital, health misinformation, corrective information, health literacy

## Abstract

Correction is an important tool to reduce the negative impact of health misinformation on social media. In the era of “I share, therefore I am” social media, users actively share corrective information to achieve the “anti-convincing” effect of health misinformation. Focusing on the local Chinese context, this study constructs a structural equation model using social capital as a mediating variable to explore whether usage of Chinese users’ social media can promote corrective information sharing by influencing the structural, cognitive, and relational dimensions of social capital and the role of health literacy in corrective information sharing. It was found that social media use did not significantly affect corrective information share willingness but significantly influenced share willingness through social interaction connections, trust, and shared experiences, and share willingness significantly influenced sharing behavior. The moderating effect showed that health literacy played a significant moderating effect in the influence of corrective information share willingness on sharing behavior. This study introduces the three dimensions of social capital at the theoretical level and finds that users will share corrective information for the purpose of social capital accumulation. It also provides empirical evidence for specific practices, including improving users’ health literacy and actively mobilizing them to participate in the blocking and management of health misinformation in social media.

## Introduction

In recent years, online users have increasingly used social media to seek and share health information (1). However, the emergence of social media has also opened the door to the proliferation of health risks, and the rapid spread of a large amount of unconfirmed and misinformation has dismantled the authenticity and scientific validity of health information (2). Health misinformation is “a claim, opinion, or content that is currently proven to be false in relation to health due to a lack of scientific evidence” (3) and may spread faster and more easily on social media than scientific information (4). The rapid proliferation of health misinformation can lead to misunderstanding and anxiety among users (5), reduces trust in health professionals, delays or hinders the adoption of individual treatment behaviors, and, in some cases, even threatens life safety (6). How to curb the spread of health misinformation on social media and defend against is an important issue concerning the health of the Chinese people.

Correction that designed to refute inaccurate claims and misleading information is an important tool to combat health misinformation. While numerous studies have recognized the efficacy of corrective messages in debunking misinformation, limited research has been conducted on Chinese samples. A considerable amount of foreign research has noted the role of correction in counteracting the proliferation of health misinformation on social media. For example, providing coherent alternative explanations for misinformation and making timely corrections can be effective in reinforcing correct information and reducing people’s misconceptions (7). Again, algorithmic correction can be used to dispel people’s misconceptions (8). Further research suggests that relying on physicians (9), experts (3), health agencies (8), and relevant authorities (10) to correct health misinformation in order to prevent backfire effects could results better.

Considering the reality of “how to correct health misinformation on social media,” the United Nations (UN) saw the “human sharing potential” and encouraged people to share real health information and correct misinformation on social media (11). In the age of “I share, therefore I am” social media, users’ likes, retweets, comments, and other sharing behaviors encourage the proliferation of health misinformation. This study focuses on whether the power of sharing can be used to achieve the “anti-convincing” effect of corrective information (12). Whether in weak or strong relationships, individuals are always a key part of reducing the spread of misinformation (13) and have great potential to correct health misinformation on social media (14).

Chinese society is a relationship-based society, where individual behavior starts with interpersonal relationships and human exchange occurs within the relationships (15). The term “relationship” in Chinese society can be included in the study of social networks and social capital. It is only that social networks emphasize the structural study of relationships, while social capital emphasizes the operation of relationships. In China, individuals’ information-sharing behaviors are not only for entertainment but also for maintaining relationships with others and acquiring social capital (16). It has been shown that the structural, relational, and cognitive dimensions of social capital all have varying degrees of influence on motivation to share personal information (17). Moreover, in the social media information interaction environment, social capital can significantly influence people’s information sharing behavior (18). For example, people will decide whether to retweet medical crowdfunding information based on favor exchange rules with the goal of gaining social capital (19). Lin believes that social capital is an important resource embedded in social networks. Then, it is worthwhile to pay attention to how to make good use of this resource in social media and construct sharing relationships with social capital as the core (20).

Based on the above discussion, this study attempts to construct an integrated model using social capital as a mediating variable to explore the factors influencing social media use and corrective information sharing. The value of this study lies in the following: first, it is based on Chinese society and considers the role of “relationship” in corrective information sharing, which expands the scope of rational behavior theory and social capital theory and adds empirical evidence. Second, given the increasing prevalence of health misinformation in social media, this study discusses how to maximize the effectiveness of corrective information in terms of specific sharing dimensions and thus provides targeted suggestions for mobilizing users to participate in corrective information sharing.

## Literature review and research hypothesis

### Social media use

Social media (social networking or Web 2.0) is a broad concept that refers to a variety of web-based platforms and services that allow users to post public or semi-public profiles and/or content and connect to other users’ profiles and/or content (21). Bolton et al. (22) believe that users can create and share a variety of contents online through the use of social media. As a result, social media use has become an essential information interaction action in citizens’ daily lives. Correa et al. (23) suggest that social media use is a special form of consumption of digital media or the Internet that is not unlike traditional media use. Within the past decade, research on social media has become a major focus of academic attention. Scholars have explored the effects of social media use on citizen participation in political life (24), information seeking and sharing behavior (25), consumer engagement (26), public perception of disease risk (S.-H (27).), worry, anxiety, and fatigue psychological mood (28) from a variety of disciplines including communication, information science, management, medical science, and psychology.

Social media use affects users’ willingness to share both positive and negative information. On the one hand, social media use can lead to the viral spread of negative information such as fake news (29), misinformation (30), and rumors (31). On the other hand, social media use can also promote the sharing of positive information such as health information (32) and corrective information (33). Because social media exacerbates the proliferation of negative information, there is tremendous value in studying the impact of social media use on positive information. Bode studied the experience of correction on social media during COVID-19 and found that most people who shared misinformation not only saw observed misinformation corrected but also potentially shared the corrective information (34). By studying how individuals deal with misinformation and corrective information about genetically modified food safety on social media, Wang found that using social media can enhance individuals’ acceptance and sharing of corrective information (35). Based on this, this study will explore the effects of social media on corrective information sharing intentions at the positive information sharing level and propose hypotheses.

This study examines the impact of social media on the willingness and behavior of corrective information sharing at the level of positive information sharing and proposes the following hypotheses:

*H1*: Social media use has a positive influence on corrective information share willingness.

Behavior and intention as important correlated variables in the theory of rational behavior and the theory of planned behavior have long been confirmed by numerous studies. Existing studies have shown that intentions effectively predict the adoption of behaviors such as health knowledge adoption (36) and social media use (37). However, it has also been shown that intention is not a significant predictor of behavior. For example, social media content may increase users’ intentions and knowledge related to Human Papilloma Virus (HPV) but do not improve behavioral outcomes (38). In the study of social media information sharing, Chen explored people’s motivation to share social crisis information through WeChat and found that there was a positive influence of willingness of WeChat users’ social crisis information sharing on behavior (16). As social crisis messages, people share corrective information to get positive comments from others, socialize, or complete their social activities. To explore what relationship actually exists between corrective information sharing intentions and behaviors, the following hypothesis was proposed:

*H2*: Corrective information sharing willingness has a positive influence on sharing behavior.

Based on the above discussion, this study asks research question Q1: Does social media use positively influence corrective information sharing behavior through corrective information sharing intentions?

### Social capital and three dimensions

As one of the first scholars to propose social capital, L.J. Hanifan defined it as “the goodwill, friendship, mutual sympathy, and social interactions among individuals and families that constitute the social unit” [(39), p. 130]. Scholars have subsequently offered different explanations for social capital, but all agree on the importance of social relationships and resources. Pierre Bourdieu considers social capital as the sum of the real or potential resources possessed by a society or group, consisting mainly of the network of relationships that define the identity of the members of the society or group [(40), p. 249]. Putnam proposed that social capital depends on the relationships between people and distinguished between bridging and bonding social capital, arguing that both forms of social capital have strong positive effects [(41), p. 23]. Nahapiet and Ghoshal (42) integrated previous research on different aspects of social capital, defined social capital as relational resources embedded in interpersonal, group, and social networks, and proposed to measure social capital in terms of structural, relational, and cognitive dimensions. Following the above framework, this study examined the influence of social capital on correct information sharing willingness using the structural dimension—social interaction ties and the cognitive dimension—shared experience and the relational dimension—trust, respectively.

### Social interaction ties

Network ties, a key concept in the structural dimension of social capital, affect both the parties that combine and exchange resources, and the expected value obtained through the exchange (42). Developed from network ties, social interaction ties refer to connections between network members that act as a medium for information flow and resource exchange and provide individuals with access to the resources of others (43). In the field of behavioral research, the more frequently social interactions are connected, the higher the intensity, depth, and frequency of information exchange, the stronger the willingness of individuals to share or contribute certain content, such as information or knowledge (44). Thus, social interaction ties are considered a key concept for measuring willingness to share and behavior.

### Trust

Trust is the core of the relational dimension of social capital (45) and is a dynamic cohesive factor that influences the achievement of the goals of both partners (46). Sociologists define trust as a set of expectations held by those involved in an exchange (47) that encompasses beliefs about the competence, integrity, and reliability of others (48). It is the existence of trust that makes it possible to maintain stable social relationships (K (49).). Research shows that trust, whether between acquaintances or strangers, leads to higher benefits and lower exchange costs for both parties to the exchange (50). Similar to face-to-face interactions, trust is also a major factor influencing online interaction behavior (51). In terms of virtual community knowledge sharing, trust involves the emotional connection between individuals and group members, which can reduce the uncertainty and risks associated with communication and coalesce identity (52) and thus inspire more information sharing behavior.

### Share experience

According to Habermas’ lifeworld theory, the cognitive dimension emphasizes culturally shared attributes and can refer to the similar attitudes, perceptions, and understandings that network members have about the “context” in which they live together (53). Common experience is the existence of similar experiences of social network members about something, and the more common points the more conducive to internal communication and cooperation, which, in turn, generates higher social capital (54). This is similar to McPherson’ formulation of “homogeneity,” i.e., people interact socially and transmit information based on the principle of homogeneity (55). Communicating with people who are different can lead to cognitive dissonance and distorted information while communicating with people who are similar tends to be more fluid and efficient (56). For example, interactions between people with similar cultures, religions, and ideologies occur more frequently than unrelated individuals (57).

## The intermediary role of social capital

### Social media use and social capital

Media interactions influence and shape interpersonal relationships, and the use of both traditional mass media and new media has positive implications for the accumulation of social capital among audiences (58). Social media, with the original intention of creating connections, not only deepens the maintenance of strong relationships but also provides a new ground for the establishment of weak relationships (59), becoming an important way for people to maintain social connections. In addition to building relationships, using social media for social interactions such as liking, commenting, and sharing can increase social capital (60). As confirmed by the study, there is a positive correlation between the usage behavior of social media users such as Instagram and WeChat to social capital (61). Specific to particular groups, such as college students (62) and older adults (63), social media use similarly shows a significant effect on social capital. This is because the longer and more frequently the medium is used, the more likely it is to engage in social capital building activities (64). Based on the above discussion, the following hypotheses are proposed:

*H3*: Social media use has a positive influence on social interaction ties of structural social capital.

*H4*: Social media use has a positive influence on trust of relational social capital.

*H5*: Social media use has a positive influence on shared experience of cognitive social capital.

### Social capital and corrective information sharing willingness

Social interaction ties can positively influence knowledge acquisition (65), as well as the quality (66) and quantity (17) of knowledge sharing. In social media contexts, social interaction ties not only increase users’ willingness to continue using WeChat (67) but also have a direct impact on social media information dissemination behavior (68). As a result, those who have good relationships and strong connections with others are more likely to share thought-provoking and valuable information, such as corrective information. Trust can encourage social media users to engage in more disclosure behaviors and share more information with trusted people (69). When trust is higher, the tendency to share information also rises (70). In addition, a study investigated the factors influencing the sharing of cancer experiences among fathers of children with cancer and found that although these fathers did not know each other, they experienced support for each other in the sharing of common cancer experiences (71). Experiencing the same negative emotional event together can promote cooperative behavior among individuals compared with experiencing negative emotional events alone (72). In addition, research on social media information sharing behavior has found that users seek out and share news and information in similar networks (73). Based on this, this study argues that people who have been exposed to or misled by health misinformation about such similar experiences are more likely to develop corrective information sharing behaviors. Moreover, the following hypotheses are proposed:

*H6*: Social interaction ties of structural social capital have a positive influence on corrective information sharing willingness.

*H7*: Trust of relational social capital has a positive influence on corrective information sharing willingness.

*H8*: Shared experience of cognitive social capital has a positive influence on corrective information sharing willingness.

At the same time, this study raises the research question Q2: What is the mediating role of social capital social interaction ties, trust, and shared experiences between social media use and corrective information sharing willingness?

### The moderating role of health literacy

Exploring health information sharing intentions and behaviors from the perspective of health literacy has become a focus of health communication research. Previous research has found that people with higher health literacy are more likely to receive more adequate health information from multiple sources. Health information literacy is positively associated with various health promotion behaviors, i.e., health literacy positively influences health information behaviors (74). Yang investigated the health information literacy of older adults and their intention to spread health rumors and found that health information literacy and the purpose of knowledge acquisition was negatively associated with the willingness of older adults to share health rumors (75). Oh and Lee confirmed the interaction between health literacy and perceived information importance in predicting willingness to verify and share health information by examining when people verify and share health rumors on social media (76). Fleary systematically reviewed and analyzed the literature on the relationship between adolescent health literacy and health behaviors. Functional and media health literacy was found to have a significant positive effect on the adoption of adolescent health behaviors (77). From the above, it can be observed that there is a significant effect of health literacy on both health intention and health behavior. However, Brittani Crook found that while health literacy positively influenced share willingness, people with higher health literacy tended to share less information about heart health than those with lower health literacy (78). Moreover, what role does health literacy play in the relationship between share willingness and behavior? To explore this question, the following hypothesis was proposed:

*H9*: Higher health literacy is associated with a stronger relationship between corrective information share willingness and behavior.

**Figure 1:**
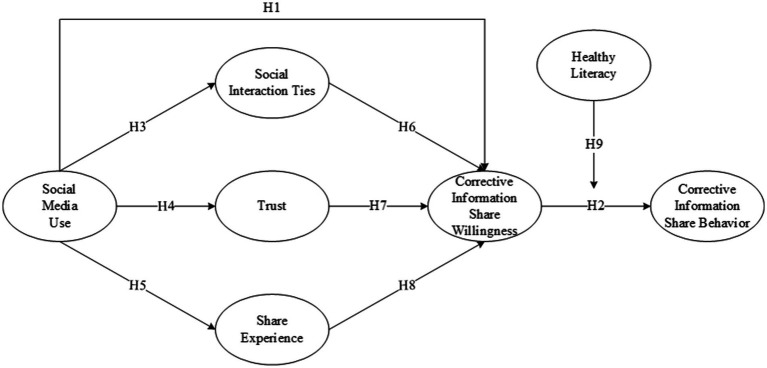
Research model for corrective information sharing in social media.

Based on the literature review, this study constructed a structural equation model of social media corrective information sharing factors (Figure 1).

## Research design

### Data collection and implementation

The survey population of this study is users who use social media in China. It mainly includes WeChat, Weibo, QQ, Zhihu, Douban, Douyin, and Kuaishou short video social media platforms. Questionnaire Star was utilized to sketch the questionnaire and distribute it via WeChat moments on 10 August 2022 and 10 October 2022. A total of 601 subjects responded to the survey, excluding 66 invalid subjects and responses. A total of 527 subjects remained, with a sample qualification rate of 87.7%. Table 1 shows the demographic information of the respondents.

**Table 1:** Demographics (number of subjects = 527).

Measure	Items	Number	Percentage(%)
Gender	Male	216	41.0
	Female	311	59.0
Education	High school or below	7	1.3
	College	42	8.0
	University	247	46.9
	Graduate school or above	231	43.8
Work	Public institutions/ Civil Servant	79	15.0
	Private enterprise	83	15.7
	State Owned Enterprises	49	9.3
	Pupil	237	45.0
	Freelancers	46	8.7
	Others	33	6.3
Age	<25 year	239	45.3
	26-35 year	170	32.3
	36-45 year	69	13.1
	46-55 year	40	7.6
	>55 year	9	1.7
Monthly Salary (RMB)	<3000RMB	233	44.2
	3,001–8,000	176	33.4
	8,001–13,000	77	14.6
	13,001–16,000	18	3.4
	>16,000	23	4.4

### Measurement development

This study contains seven measurement dimensions, six of which were designed with options using a seven-point Likert scale (“1–7” for “strongly disagree” to “strongly agree”). to ensure the reliability and validity of the questionnaire, a small-scale test was conducted on the subjects in the pre-testing stage to listen to their opinions, and the questions, statements, and wording of the relevant questions were modified. After that, we invited experts and scholars to review the questionnaire and gradually revised it to improve it. The variables, measurements, and sources are shown in Table 2.

**Table 2:** Summary of measurement scales.

Construct	Measure	Source
Social Media Use(SMU)		
SMU1	Number of times you use SM per day	(67)
SMU2	Number of chats with others in SM per day	
SMU3	Number of times you retweet content from SM per day	
Social Interaction Ties(SIT)		
SIT1	I have close relationships with SMM	(43, 79)
SIT2	I spend a lot of time interacting with SMM	
SIT3	I have frequent communication with SMM	
SIT4	I have established a steady connection with SMM	
Trust(TRU)		
TRU1	My SMM are sincere with each other	(43, 79)
TRU2	My SMM do not try to use people in any way	
TRU3	My SMM keep their promises to each other	
TRU4	My SMM do not interrupt people’s conversations with malicious intent	
TRU5	My SMM are consistent with their words	
Share Experience(SHE)		
SHE1	My SMM and I have had the experience of being misled by HM	(70, 80)
SHE2	My SMM and I have similar views on HM	
SHE3	My SMM and I have similar attitudes to HM	
SHE4	My SMM and I handle HM in a similar way	
Corrective Information Share Willingness(CISW)		
CISW1	I want to share CI to others	(81, 82)
CISW2	I wish to share CI with others	
CISW3	I look forward to sharing CI with others	
CISW4	I will continue to share CI to others	
Corrective Information Share Behavior(CISB)		
CISB1	Number of times per week I have shared CI with my family in the last three months	(17, 83)
CISB2	Number of times per week I have shared CI with friends in the last three months	
CISB3	Number of times per week I have shared CI with colleagues in the last three months	
CISB4	Number of times per week I have shared CI with others in the last three months	
Health Literacy(HEL)		
HEL1	I know where to seek health information	(84, 85)
HEL2	I like to get health information from diverse sources	
HEL3	Assessing the reliability of health information on diverse websites is easy for me	
HEL4	Assessing the reliability of health information on social media is easy for me	
HEL5	I apply diverse health knowledge to my daily life	

SM, Social Media; SMM, Social Media Members; HM, Health Misinformation; CI, Corrective information.

### Statistical analysis and hypothesis test

This study adopted SPSS23.0 for descriptive analyses, and Partial Least Square (PLS) was used for confirmatory factor analyses and research hypotheses testing. PLS is considered “the most complete and versatile system” in structural equation modeling (86), and PLS-SEM can be used for principal component analysis, path analysis, testing for mediation and moderating effects and produces more robust results for non-constant data situations. The structural equation model evaluation and analysis for this study were carried out using SmartPLS 4.0.

### Measurement model

The measurement model must pass the reliability test before structural model analysis can be performed. According to the statistical test, the standardized factor loading (STD) of the measurement model should be higher than 0.50, the composite reliability (C.R.) higher than 0.60, and the average variance extracted (AVE) higher than 0.50. As shown in Table 3, the STD of all items is greater than 0.8, Cronbach’s alpha and C.R. are greater than 0.8, and AVE is greater than 0.7, all of which meet the criteria suggested by scholars and prove that all variables and items in the measurement model and question items have good reliability and validity.

**Table 3:** Reliability and convergent of the research model.

Variables	Items	Factor Loadings	Cronbachs α	C.R.	AVE
SMU	SMU1	0.924	0.907	0.918	0.842
	SMU2	0.912			
	SMU3	0.916			
SIT	SIT1	0.908	0.931	0.932	0.828
	SIT2	0.902			
	SIT3	0.926			
	SIT4	0.903			
TRU	TRU1	0.882	0.939	0.942	0.804
	TRU2	0.899			
	TRU3	0.909			
	TRU4	0.907			
	TRU5	0.886			
SHE	SHE1	0.828	0.896	0.903	0.762
	SHE2	0.887			
	SHE3	0.917			
	SHE4	0.857			
CISW	CISW1	0.929	0.953	0.953	0.876
	CISW2	0.944			
	CISW3	0.935			
	CISW4	0.935			
CISB	CISB1	0.929	0.952	0.953	0.874
	CISB2	0.944			
	CISB3	0.928			
	CISB4	0.938			
HEL	HEL1	0.817	0.902	0.914	0.719
	HEL2	0.812			
	HEL3	0.890			
	HEL4	0.900			
	HEL5	0.816			

C.R., composite reliability; AVE, average variance extracted; SMU, Social Media Use; SIT, Social Interaction Ties; TRU, Trust; SHE, Share Experience; CISW, Corrective information share Willingness; CISB, Corrective information Share Behavior; HEL, Health Literacy.

Average variance extracted, as an important indicator to test whether variables are distinguishable, is crucial to the construction of models and the success of research hypotheses. In this study, the international Fornell–Larcker criterion was used to measure the discriminant validity between variables (87). As shown in Table 4, the square root of AVE of the variables in the measurement model is greater than the correlation coefficient between the variables, indicating that all variables have good discriminant validity between them.

**Table 4:** Discriminant validity of measurement models (AVE).

Variables	AVE	SMU	SIT	TRU	SHE	CISW	CISB
SMU	0.842	0.918					
SIT	0.828	0.286	0.910				
TRU	0.804	0.203	0.611	0.897			
SHE	0.762	0.171	0.476	0.521	0.873		
CISW	0.876	0.156	0.448	0.465	0.514	0.936	
CISB	0.874	0.103	0.209	0.155	0.321	0.344	0.935

The diagonal values are the square root of each variable AVE and the others are the correlation coefficients between the variables.

## Structural model

There are four main model fit metrics in PLS-SEM are standardized root mean square residual (SRMR), bootstrap-based test for the exact overall model fit (d_ULS and d_G),^1^ and normed fit index (NFI). In this study, SRMR = 0.038 (<0.08), d_ULS = 0.437(<0.95), d_G = 0.327 (<0.95), and NFI = 0.911(>0.90) all meet the PLS model fitting standards recommended by scholars (88, 89). Therefore, the model of this study has a good degree of fit and can be analyzed in the next step.

### Path analysis and hypothesis testing

As shown in Table 5, varying degrees of support for all seven research hypotheses, except for social media use, on corrective information sharing willingness did not receive support. There was a positive and significant effect of corrective information sharing willingness (CISW) (*β* = 0.173, *p* < 0.001) on corrective information sharing behavior (CISB). Social media use (SMU) (*β* = 0.286, *p* < 0.001) significantly influenced social interaction ties (SIT), social media use (SMU) (*β* = 0.203, *p* < 0.001) significantly influenced trust (TRU), and social media use (SMU) (*β* = 0.171, *p* < 0.001) significantly influenced share experience (SHE). There was a positive and significant effect of social interaction ties (SIT) (*β* = 0.175, *p* = 0.012), trust (TRU) (*β* = 0.182, *p* = 0.007), and share experience (SHE) (*β* = 0.334, *p* < 0.001) on corrective information sharing willingness (CISW). Therefore, the research hypothesis H1 is not valid and H2, H3, H4, H5, H6, H7, and H8 are valid.

**Table 5:** Path analysis and hypothesis test.

Hypothesis	Path	Unstd.	Std.	*p*-value	Results
H1	SMU → CISW	0.016	0.012	0.761	Reject
H2	CISW→CISB	−0.325	0.173^***^	0.000	Accept
H3	SMU → SIT	0.333	0.286^***^	0.000	Accept
H4	SMU → TRU	0.206	0.203^***^	0.000	Accept
H5	SMU → SHE	0.171	0.171^***^	0.000	Accept
H6	SIT→CISW	0.194	0.175^*^	0.012	Accept
H7	TRU → CISW	0.236	0.182^**^	0.007	Accept
H8	SHE→CISW	0.427	0.334^***^	0.000	Accept

Unstd., Unstandardization coefficient; Std., Standardization coefficient; *p < 0.05; **p < 0.01; ***p < 0.001.

### Mediation effect test

In this study, the PLS-Bootstrapping 5,000 was used to test the mediating effect. As shown in Table 6, the mediation effect of corrective information sharing willingness between social media use and corrective information sharing behavior (*β* = 0.002, *p* = 0.769) was not significant, answering Q1. Among the simple mediation effects, social interaction ties (*β* = 0.050), trust (*β* = 0.037), and share experience (*β* = 0.057) each played a significant mediating effect between social media use on corrective information sharing willingness, answering Q2. In addition, this study also found that social interaction ties (*β* = 0.030), trust (*β* = 0.032), and share experience (*β* = 0.058) each positively influenced corrective information sharing behavior through corrective information sharing willingness.

**Table 6:** Mediation effect test.

Intermediate Path	Indirect effect	*T*-value	*P*-value	Bias-corrected 95%		Results
				Lower bound	Upper bound	
SMU → CISW→CISB	0.002	0.294	0.769	−0.021	0.031	Reject
SMU → SIT→CISW	0.050^*^	2.349	0.019	0.011	0.095	Accept
SMU → TRU → CISW	0.037^*^	2.277	0.023	0.010	0.073	Accept
SMU → SHE→CISW	0.057^**^	3.056	0.002	0.025	0.098	Accept
SIT→CISW→CISB	0.030^*^	2.097	0.036	0.013	0.112	Accept
TRU → CISW→CISB	0.032^*^	2.351	0.019	0.017	0.111	Accept
SHE→CISW→CISB	0.058^**^	3.115	0.002	0.071	0.169	Accept
SMU → SIT→CISW→CISB	0.009^*^	1.965	0.049	0.004	0.034	Accept
SMU → TRU → CISW→CISB	0.006^*^	2.028	0.043	0.003	0.026	Accept
SMU → SHE→CISW→CISB	0.010^*^	2.192	0.028	0.008	0.036	Accept

*p < 0.05; **p < 0.01; ***p < 0.001.

In the chain mediation effects, social media use through social interaction ties, trust, share experience, and corrective information sharing willingness played a weak role in influencing corrective information sharing behavior. The chain mediation effect through share experience had the largest effect (*β* = 0.010).

### Moderating effect of healthy literacy

**Figure 2:**
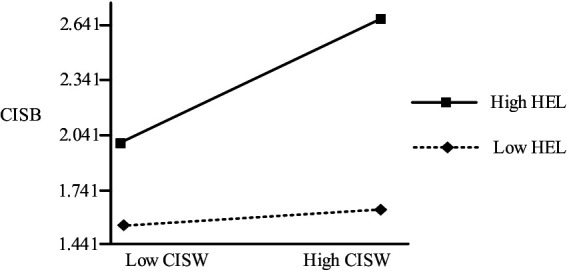
Moderating effect interaction plot of healthy literacy.

As shown in Table 7, the interaction term between share willingness and health literacy had a positive and significant effect on sharing behavior (*p* < 0.01). When health literacy is higher, the degree of influence of share willingness on behavior is stronger. Therefore, for every 1 unit increase in health literacy, the degree of influence of willingness on behavior will increase by 0.131 units (Figure 2).

**Table 7:** Moderating effect of healthy literacy.

Path	Path Factor	Standard error	*T*-value	*P*-value	Result
CISW→CISB	0.175^***^	0.042	4.206	0.000	Accept
HEL → CISB	0.345^***^	0.042	8.129	0.000	
CISW×HEL → CISB	0.131^**^	0.048	2.720	0.006	

*p < 0.05; **p < 0.01; ***p < 0.001.

### Conclusion and discussion

This study developed a structural equation model with social capital as a mediating variable and health literacy as a moderating variable. It is used to predict whether social media use can construct social capital through relational connection between users to promote the dissemination of corrective information to combat health misinformation.

## Conclusion

In terms of direct effects, first, social media use positively influence social interaction ties, trust, and share experiences in social capital. This result demonstrates that use social media can enhance an individual’s status, resources (social interaction ties), and interpersonal relationships (trust) within the social network structure and can increase the sense of identity (share experiences) among other members. In the structural dimension, social media use can maintain the communication and sharing of information, knowledge, and experience, such as the dissemination of corrective information. In the relational dimension, social media use can develop positive and good interpersonal relationships, such as affectionate relationships, friendship relationships, and trust relationships. In the cognitive dimension, social media use can enhance the level of understanding among members and share common aspirations, goals, and experiences. Second, social capital positively and significantly affects the corrective information sharing willingness. In terms of social interaction ties, the more frequent the interaction between social media members, the deeper the connections are. The more information is shared, the more knowledge and experience is exchanged, such as the sharing of corrective information. In terms of trust, the higher the level of trust among social media members, the greater the willingness to generate knowledge and information exchange, so trust can significantly influence the corrective information sharing willingness. In terms of share experience, when social media members identify with each other, the opportunity for information exchange is increased. For example, when social media members have experienced being misled by health misinformation, this experience increases the motivation of individuals to share corrective information. Third, corrective information sharing intention has a positive and significant effect on sharing behavior. It proves that when social media users generate willingness, they may generate behaviors.

In terms of indirect effects, first, social capital of social interaction ties, trust, and shared experience played a significant mediating effect on social media use to corrective information sharing willingness. It is noteworthy that social media use has no direct effect on share willingness but has an indirect effect on it through the mediating variable of social capital. The social capital of social ties, trust, and share experiences were shown to influence people’s corrective information share willingness and behavior by building social network relationships. Second, corrective information share willingness mediated the effect of social capital of social interaction ties, trust, and share experience on corrective information sharing behavior. Third, the mediating effect of share willingness on social media use to sharing behavior was not significant.

In terms of moderating effect, health literacy plays a positive moderating role between corrective information share willingness and behaviors. Therefore, it can be understood that health literacy increases the influence of corrective information share willingness on behavior, which is more conducive to the proliferation of corrective information and helps to reduce the negative impact of health misinformation.

## Discussion

### Theoretical contributions

The impact of social media use on social capital has been demonstrated in many studies covering a wide range of disciplines, including news media, sociology, psychology, education, and economics. In this study, social media use was used as the independent variable, and the interactive connection, trust, and common experience of social capital were used as the mediator variables, and health literacy was added as a moderator variable to explore the sharing behavior of corrective health information, which expands the scope of application of social capital theory. First, the results of this study respond to previous research on social media use for social interaction connections (63, 90, 70). This suggests that social media use promotes interactive connection relationships among members, which facilitates the generation of information flow and exchange. Second, in terms of trust, distinguishing from previous study, this study found that social media use could positively influence the level of trust among members. In virtual social relationships, due to frequent communication and interaction between network members, social media use allows them to share more information, thus continuously increasing the level of trust between each other. This trust formed by continuous connection can significantly gather social capital in network relationships, and at the same time, social capital will be continuously expanded due to the deepening of trust among network members. Finally, in terms of share experiences, some studies have found that media use enhances an individual’s identity and local identification, resulting in similarities with other members, such as the same background, the same context (91). Social media use can form stable networks of relationships and maintain positive and stable connections within the network with people who share common values and ideas and promote common interests (92).

This study confirms that social interaction ties, trust, and shared experiences have a significant effect on corrective information share willingness. Similar to the finding by Chiu, social interaction ties and share experiences can significantly influence individuals’ information sharing behavior (17). Furthermore, consistent with the finding by Chen, trust significantly affects corrective information share willingness (16). In terms of structural dimensions, social interaction ties, as an important channel for information and resource flow, have an important role in correcting the wrong effects of health misinformation. Second, in terms of the relationship dimension, trust had a positive and significant effect on the corrective information share willingness, echoing the findings by Chen. Voluntary-focused trust behaviors are particularly important for exploring social media users’ corrective information share willingness. Finally, to some extent, share experiences are reflected in homogeneity among social media members, i.e., whether they have all been exposed to health misinformation or whether their attitudes and perceptions about health misinformation are consistent with other members. Individuals are more likely to interact with members with whom they have something in common and are more likely to engage in corrective information sharing behavior when they believe that social media members may have similar experiences or encounters with them.

Although the findings confirm a positive and significant effect of share willingness on sharing behavior, the extent of the effect is not high. Information sharing is the act of information exchange and collaboration between two parties with a connected relationship, based on individual interests or common interests. The occurrence of behavior is influenced not only by intention but also by many factors such as individual ability, motivation, habit, cost, and convenience (93). First, information sharing requires certain costs, such as time, energy, and even privacy, to maintain an active state of communication and discussion with those being shared. Second, corrective information sharers also need to “gatekeep” the information to determine whether it is of good quality, and if they have difficulty ensuring the quality of the information, they may hesitate to share it. Especially when corrective information is published after health misinformation, many corrective information is not strictly verified, which not only does not help to correct health misinformation but also increases people’s false beliefs. Third, information sharing requires certain resources and environmental conditions for the sharers, such as network conditions and device sensitivity. Therefore, future research needs to focus on how to stimulate users’ willingness so as to cultivate and maintain their sharing behavior.

### Correction measures in China’s relational society

There have been many studies pointing to correction as an important means of addressing misinformation. In previous studies, scholars have attempted to reform the operation of news organizations in social networks to correct misinformation through fact-checking recommendations, information warnings, and growing a team of fact-checkers (94). However, this study argues that the spread of misinformation is shaped by social networks, and that to address misinformation, any corrective measures need to take social and interpersonal factors into account, or they may not achieve the corrective purpose at all. In a relational society, individuals’ behavior starts with relationships, and information is often shared and interacted with the purpose of exchanging benefits and constructing social capital. First, relationships in social capital become an important variable in predicting corrective information sharing. Psychologists believe that the three main motivations for people to create and spread rumors are to discover facts and expand interpersonal relationships and self-improvement (95). Similar to the motivation of rumor spreading, the spread of corrective information is also aimed at increasing mutual understanding and maintaining relationships. To enhance the exchange of information and benefits with members, individuals often share information in order to promote lasting relationships. Second, social capital connections are important for predicting the spread of corrective information. People often rely on informal relationships and word-of-mouth to obtain advice about health, especially when formal sources of information are not trusted. Thus, when members of a social network are subjected to health misinformation, individuals will communicate with other members and build connections and trust. In addition, the closeness of the relationship affects the persuasive effect of corrective messages, corrections from close people being more acceptable than strangers. Therefore, facilitating connections and interactions among social media members becomes a key element in the dissemination of corrective messages.

### The importance of health literacy

The “Health China 2030” plan clearly states that “the health literacy level of the population will reach 30% by 2030. Among them, health literacy is mainly reflected in the screening and understanding of health information by individuals, as well as at the level of individuals’ perception of whether health information will be threatened, their concern for health information protection, and the adoption of protective behaviors. As an important finding of this study, health literacy was a positive predictor of increased individual corrective information sharing behavior. When health literacy is higher, individuals have greater motivation and ability to transform their intentions into behaviors and thus take actual actions to convey authentic information. Therefore, developing public health literacy not only enhances individuals’ health knowledge and skills but also their behavioral intention to corrective information forwarding and spreading. On the one hand, individuals should enhance their consciousness of protection in their daily use of the Internet and try not to spread or proliferate information with uncertainty. On the other hand, individuals should actively participate in online health literacy training to improve their health literacy.

This study has some shortcomings. The assessment of health literacy was derived from self-reported data from social media users, which may overestimate the results of health literacy. In addition, follow-up studies should focus on the key role that medical experts, healthcare workers, and health agencies play in addressing health misinformation in the Chinese cultural context and which types of corrective information have better corrective effects.

## Data availability statement

The original contributions presented in the study are included in the article/supplementary material, further inquiries can be directed to the corresponding author.

## Ethics statement

Ethical approval was not required for the study involving humans in accordance with the local legislation and institutional requirements. The studies were conducted in accordance with the local legislation and institutional requirements. Written informed consent to participate in this study was not required from the participants in accordance with the national legislation and the institutional requirements.

## Author contributions

BF: Conceptualization, Data curation, Investigation, Methodology, Software, Writing – original draft, Writing – review & editing.

## Footnotes

^1^The bootstrap-based test for the exact overall model fit tests the statistical (bootstrap-based) inference of the discrepancy between the empirical covariance matrix and the covariance matrix implied by the composite factor model. d_ULS (i.e., the squared Euclidean distance) and d_G (i.e., the geodesic distance) represent two different ways to compute this discrepancy.
